# Presynaptic mGlu1 Receptors Control GABA_B_ Receptors in an Antagonist-Like Manner in Mouse Cortical GABAergic and Glutamatergic Nerve Endings

**DOI:** 10.3389/fnmol.2018.00324

**Published:** 2018-09-18

**Authors:** Matteo Vergassola, Guendalina Olivero, Francesca Cisani, Cesare Usai, Simone Bossi, Aldamaria Puliti, Anna Pittaluga

**Affiliations:** ^1^Department of Pharmacy, University of Genoa, Genoa, Italy; ^2^Institute of Biophysics, National Research Council, Genoa, Italy; ^3^Department of Neurosciences, Rehabilitation, Ophthalmology, Genetics, Maternal and Child Health, University of Genoa, Genoa, Italy; ^4^IRCCS Istituto Giannina Gaslini, Genoa, Italy; ^5^Center of Excellence for Biomedical Research, University of Genoa, Genoa, Italy

**Keywords:** mGlu1 receptor, GABA_B_ receptor, receptor–receptor interaction, *Grm1^crv4/crv4^* mice, release

## Abstract

Mouse cortical GABAergic synaptosomes possess presynaptic inhibitory GABA_B_ autoreceptors. Accordingly, (±)baclofen (3 μM) inhibits in a CGP53423-sensitive manner the 12 mM KCl-evoked release of preloaded [^3^H]GABA. Differently, the existence of presynaptic release-regulating metabotropic glutamate type 1 (mGlu1) heteroreceptors in these terminals is still matter of discussion, although confocal microscopy unveiled the existence of mGlu1α with GABA_B1_ or GABA_B2_ proteins in cortical VGAT-positive synaptosomes. The group I mGlu agonist 3,5-DHPG failed to modify on its own the 12 mM KCl-evoked [^3^H]GABA exocytosis from cortical nerve endings, but, when added concomitantly to the GABA_B_ agonist, it significantly reduced the 3 μM (±)baclofen-induced inhibition of [^3^H]GABA exocytosis. Conversely, the mGlu1 antagonist LY367385 (0.03–1 μM), inactive on its own on GABA exocytosis, amplified the 3 μM (±)baclofen-induced inhibition of [^3^H]GABA overflow. The ( ± )baclofen-induced inhibition of [^3^H]GABA exocytosis was more pronounced in cortical synaptosomes from *Grm1^crv4/crv4^* mice, which bear a spontaneous mutation of the *Grm1* gene leading to the functional inactivation of the mGlu1 receptor. Inasmuch, the expression of GABA_B2_ receptor protein in cortical synaptosomal lysates from *Grm1^crv4/crv4^* mice was increased when compared to controls. Altogether, these observations seem best interpreted by assuming that mGlu1 coexist with GABA_B_ receptors in GABAergic cortical synaptosomes, where they control GABA receptors in an antagonist-like manner. We then asked whether the mGlu1-mediated control of GABA_B_ receptors is restricted to GABAergic terminals, or if it occurs also in other subpopulations of nerve endings. Release-regulating GABA_B_ receptors also exist in glutamatergic nerve endings. (±)baclofen (1 μM) diminished the 12 mM KCl-evoked [^3^H]D-aspartate overflow. Also in these terminals, the concomitant presence of 1 μM LY367385, inactive on its own, significantly amplified the inhibitory effect exerted by (±)baclofen on [^3^H]D-aspartate exocytosis. Confocal microscopy confirmed the colocalization of mGlu1 with GABA_B1_ and GABA_B2_ labeling in vesicular glutamate type1 transporter-positive particles. Our results support the conclusion that mGlu1 receptors modulate in an antagonist-like manner presynaptic release-regulating GABA_B_ receptors. This receptor–receptor interaction could be neuroprotective in central disease typified by hyperglutamatergicity.

## Introduction

Dimerization of G protein-coupled receptors (GPCRs) is a necessity for signal transduction, leading from agonist binding to G protein activation. Homodimers originate from the association of two units of single receptor proteins, while heterodimers involve different receptor proteins. Metabotropic glutamate (mGlu) receptors exist as either homo or heterodimers (Doumazane et al., 2010; Nicoletti et al., 2011), while GABA_B_ receptors are heterodimers (Pin and Bettler, 2016).

GABA_B_ receptors have a widespread distribution in the central nervous system (CNS) where they mediate the inhibition of chemical transmission. They preferentially locate presynaptically, close to the site of transmitter release, and contribute to control synaptic plasticity. GABA_B_ receptors exist as auto-receptors on GABAergic nerve terminals (Pittaluga et al., 1987) and as heteroreceptors on non-GABAergic terminals (i.e., the glutamatergic and the peptidergic nerve endings, Bonanno and Raiteri, 1993).

Release-regulating mGlu1 receptors also locate presynaptically in CNS (Pittaluga, 2016) where they control glutamate (Musante et al., 2008) noradrenaline (Longordo et al., 2006) and acetylcholine (Feligioni et al., 2003) release. mGlu1 receptor proteins are largely expressed in GABAergic interneurons. In particular, evidence in the literature demonstrate that the mGlu1 receptor protein exists in GABAergic neurons in the cortex, in the striatum, in the hippocampus and in the cerebellum (Pellegrini-Giampietro, 2003; Ferraguti et al., 2008). The effects that follow mGlu1 receptors activation/inactivation suggest they could have a main role in controlling synaptic plasticity (Battaglia et al., 2001; Pellegrini-Giampietro, 2003). Agonist acting at mGlu1 receptors depress synaptic transmission in the CA1 region of the rat hippocampus (Gereau and Conn, 1995; Morishita et al., 1998), while, in rat corticostriatal slices, it inhibits GABA-mediated inhibitory postsynaptic currents (Battaglia et al., 2001). Because of these actions, antagonists acting at mGlu1 receptors are propose to be neuroprotective (see for a review Pellegrini-Giampietro, 2003). Clear evidence of the presynaptic release-regulating activity of mGlu1 receptors in GABAergic nerve terminals, however, are so far incomplete and deserve further investigation. 3,5-DHPG was reported to increase the spontaneous GABA release from rat parietal-cortical synaptosomes (Bragina et al., 2015), but it failed to affect the release of GABA elicited by a mild depolarizing stimulus from mouse cortical and hippocampal GABAergic nerve endings (Musante et al., 2010; Rossi et al., 2013; Zucchini et al., 2013; Pittaluga, 2016).

Evidence in the literature evidentiate that mGlu1 and GABA_B_ receptor proteins are co-expressed and physically associate in selected CNS regions (Ige et al., 2000; Tabata et al., 2004; Luján and Shigemoto, 2006; Rives et al., 2009; Tadavarty et al., 2011). Furthermore, activation of GABA_B_ receptors was shown to increase calcium responses generated by mGlu1 receptors, consistent with the functional cross-talk of the two GPCRs (Hirono et al., 2001; Tabata et al., 2004). Conversely, whether mGlu1 receptors could affect GABA_B_-mediated responses was not so far investigated.

The present study aimed at confirming the existence of mGlu1 heteroreceptors in cortical GABAergic nerve endings, and, concomitantly, at highlighting if these receptors could influence the release-regulating activity of colocalized presynaptic GABA_B_ autoreceptors. Based on previous observations showing that mGlu1 receptors could not modify *on its own* the depolarization-evoked release of preloaded [^3^H]GABA, we posited that cortical synaptosomes could represent an appropriate model to highlight the mGlu1/GABA_B_ receptor–receptor interaction. The working hypothesis is that, if present in GABAergic nerve endings, the activation of presynaptic mGlu1 receptors could elicit an intra-terminal cascade of events *insufficient* “*per se*” to alter GABA exocytosis, but *sufficient* to modulate intraterminal processes which may affect the functions of other proteins, including the GABA_B_ subunits (Longordo et al., 2006). The study was also extended to glutamatergic terminals to investigate whether mGlu1 receptor modulates presynaptic release-regulating GABA receptors also in non-GABAergic terminals.

## Materials and Methods

### Animals

Mice (male, strain C57BL/6J) were obtained from Charles River (Calco, Italy) and were housed in the animal facility of DIFAR, Pharmacology and Toxicology Section, under environmentally controlled conditions (temperature = 22°C, humidity = 40%) on a 12-h light/dark cycle with food and water *ad libitum*. Breeding procedures were in accordance with the European legislation (European Communities Council Directive of 24 November 1986, 86/609/EEC) and the ARRIVE guidelines.

*Grm1^crv4^* mice with the spontaneous recessive *crv4* mutation were also used. The *crv4* mutation occurred in the BALB/c/Pas inbred strain and consisted of an intronic insertion of a retrotransposon LTR (Long Terminal Repeat) fragment that disrupted the *Grm1* gene splicing, causing the absence of mGlu1 receptor protein. *Grm1^crv4/crv4^* homozygous mice presented mainly with motor coordination deficits and bone defects (Conti et al., 2006; Musante et al., 2017). Affected (*Grm1^crv4/crv4^*) and control [*Grm1^+/+^, wild type (WT)*] mice were maintained on the same genetic background by intercrossing *Grm1^crv4/+^* mice. The animals were housed at the animal facility of the IRCCS A.U.O. San Martino-IST (Genoa, Italy). The procedures for breeding and genotyping of *Grm1^crv4/crv4^* mice were reviewed and approved by the Animal welfare ethical committee of the IRCCS-AOU San Martino-IST National Cancer Research Institute (Genoa, Italy), and definitive approval obtained by the Italian Ministry of Health (DDL 26/2014 and previous legislation; protocol number 371). To obtain the genotype of the mouse progeny, DNA was extracted from ear clippings according to the manufacturer’s protocol (KAPA Mouse Genotyping Kits). *Crv4* mutation was detected by DNA polymerase chain reaction (PCR) amplification using specific primers as previously described (Musante et al., 2010; Rossi et al., 2013).

All the mice were euthanized by cervical dislocation, followed by decapitation, and the cortices were rapidly removed. The experimental procedures were carried out at the animal facility of DIFAR, Pharmacology and Toxicology Section, and approved by the Italian Ministry of Health (DDL 26/2014 and previous legislation; protocol number 02/10/06/2015-OPBA), according to the Guidelines for Animal Care and Use of the National Institutes of Health and according to the Society’s Policies on the Use of Animals and Humans in Neuroscience Research. In line with the 3Rs rules (replacement, refinement, and reduction), any effort was made to reduce the number of animals to obtain statistically reliable results. All experiments were performed using adult animals (3–8 months of age).

### Preparation of Synaptosomes

Mouse cortical purified synaptosomes were prepared as previously described (Summa et al., 2013). Briefly, the mouse cortex was homogenized in 10 volumes of 0.32 M sucrose, buffered to pH 7.4 with Tris-(hydroxymethyl)-amino methane (TRIS, final concentration 0.01 M) with a glass/Teflon tissue grinder (clearance 0.25 mm). The homogenate was centrifuged (1000 x *g* for 5 min) to remove nuclei and debris, and the supernatant was gently layered on a discontinuous Percoll gradient (6, 10, and 20% v/v in Tris-buffered sucrose). After centrifugation at 33,500 x *g* for 5 min, he layer between 10 and 20% Percoll (synaptosomal fraction) was collected and washed by centrifugation (20,000 × g for 16 min). Synaptosomes were resuspended in a physiological medium having the following composition (mM): NaCl, 140; KCl, 3; MgSO_4_, 1.2; CaCl_2_, 1.2; NaH_2_PO_4_, 1.2; NaHCO_3_, 5; HEPES, 10; glucose, 10; pH 7.4.

### Release Experiments

Synaptosomes were incubated for 15 min at 37°C in a rotary water bath in the presence of [^3^H]GABA (f.c: 20 nM) or [^3^H]D-aspartate ([^3^H]D-Asp, f.c.: 50 nM). Fifty micrometer amino-oxyacetic acid was added during the incubation to avoid GABA catabolism. Identical portions of the synaptosomal suspension were then layered on microporous filters at the bottom of parallel thermostated chambers of a Superfusion System (Raiteri et al., 1974; Pittaluga, 2016; Ugo Basile, Comerio, Varese, Italy). Synaptosomes were then superfused at 0.5 ml/min with physiological medium. Synaptosomes were equilibrated during 36 min of superfusion and starting from *t* = 36 min superfusate fractions were collected as follows to quantify tritium release: two 3-min samples (basal release), one before (*t* = 36–39) and one after (*t* = 45–48 min) a 6-min fraction (*t* = 39–45 min; evoked release). Synaptosomes were exposed for 90 s, starting from *t* = 39 min, to high KCl solution (12 mM, NaCl substituting for an equimolar concentration of KCl, Zucchini et al., 2013), in the absence or in the presence of GABA_B_ receptor and/or mGlu1 receptor agonists and antagonists, as well as protein kinase C (PKC) inhibitor.

The amount of radioactivity released into each superfusate fraction was expressed as percentage of the total synaptosomal radioactivity. The 12 mM KCl -evoked tritium overflow was evaluated by subtracting the neurotransmitter content in the first and in the third fractions collected (basal release, b1 and b3) from that in the 6-min fraction collected during and after the depolarization pulse (evoked release, b2). In all the figures, data are reported as the mean ± SEM of independent determinations obtained in different experiments run in triplicate (at least three superfusion chambers for each experimental condition).

### Confocal Microscopy and Colocalization

Mouse cortical synaptosomes were fixed with 2% paraformaldehyde, permeabilized with 0.05% Triton X-100 phosphate-buffered saline (PBS) and incubated overnight at 4°C with the following primary antibodies diluted in 3% albumin PBS: rabbit anti-mGlu1a (1:500), mouse anti-GABA_B1_ (1:500), mouse anti-GABA_B2_ (1:500), guinea pig anti-vesicular GABA transporter (VGAT, 1:300), and guinea pig anti-vesicular glutamate transporter type 1 (VGLUT1; 1:500) as indicated. Synaptosomes were then washed in PBS and incubated for 1 h at room temperature with the following secondary antibodies: donkey anti-mouse AlexaFluor-647, goat anti-guinea pig AlexaFluor-488, goat anti-rabbit AlexaFluor-555 as appropriate. Finally, synaptosomes were applied onto coverslips (Musante et al., 2008). Fluorescence images (512 × 512 pixels) were acquired by a Leica TCS SP5 confocal microscope, through a 63X/1.4 NA objective. Bleed-through of emission spectra was avoided by sequential channel acquisition. The evaluation of colocalized proteins was performed as previously described (Summa et al., 2013), by using the “Colocalization threshold” plugins (WCIF Colocalization Plugins, Wright Cell Imaging Facility, Toronto Western Research Institute, Canada) in the ImageJ 1.51w software (Wayne Rasband, NIH, Bethesda, MD, United States).

### Immunoblot Analysis

Cortical synaptosomes from *Grm1^crv4/crv4^* and WT mice of the same breeding and age were homogenized in lysis buffer [10 mM Tris, pH 8.8, 20% glycerol, 2% sodium dodecyl sulfate (SDS), 0.1 mM Ethylenediaminetetraacetic acid (EDTA), 5% β-mercaptoethanol]. The protein concentration of the homogenates was determined using the Bradford method. Twenty microgram of total protein was separated on a 4–15% precast polyacrylamide gel (Bio-Rad) by means of SDS–polyacrylamide gel electrophoresis. The concentration of proteins in each sample was on the linear portion of the curve. A triplicate analysis was performed for each lysate sample. Electroblotted proteins were monitored using Naphtol blue black staining. Membranes were then incubated with the following antibodies: mouse monoclonal anti-mGlu1 receptor antibody (1:2500); mouse monoclonal anti-GABA_B1_ receptor antibody (1:500); mouse monoclonal anti-GABA_B2_ receptor antibody (1:500); mouse monoclonal anti-Gapdh antibody (1:10000). After incubation with peroxidase-coupled secondary antibodies, protein bands were detected by using a Western blotting detection system (ECL Advance^TM^). Bands were detected and analyzed for density using an enhanced chemiluminescence system (Versa-Doc 4000; Bio-Rad), and QuantityOne software (Bio-Rad). All of the protein bands used were normalized for Gapdh level in the same membrane.

### Statistical Analysis

For data handling/statistics and for graph drawing Sigma plot 10 data analysis and graphing software package was used. Analysis of variance was performed by ANOVA, followed by Dunnett’s or Tukey’s multiple-comparisons test; direct comparisons were performed by Student’s *t*-test or by Mann Whitney test, as indicated. The level of significance was set at *p* < 0.05.

### Chemicals

[2,3-^3^H]D-aspartate (specific activity 11.3 Ci/mmol) and [^3^H]GABA (specific activity 30.0 Ci/mmol) were from Perkin Elmer (Boston, MA, United States). (±)-baclofen, LY367385, (RS)-3,5 DHPG, and CGP 52432 were purchased from Tocris Bioscience (Bristol, United Kingdom). GF109203X, aminooxyacetic acid, naphtol blue black staining, horseradish peroxidase-coupled anti-mouse and anti-rabbit secondary antibodies were from Sigma (Milan, Italy). Donkey anti-mouse AlexaFluor-647, goat anti-guinea pig AlexaFluor-488, goat anti-rabbit AlexaFluor-555 were from Life Technologies Corporation (Carlsbad, CA, United States). Mouse anti-GABA_B1_ and mouse anti-GABA_B2_ antibodies were from Santa Cruz Biotechnology (Dallas, TX, United States). Rabbit anti-mGlu1 antibody, used in confocal analysis, was from Abcam (Cambridge, United Kingdom) and mouse anti-mGlu1 monoclonal antibody, used in Western blotting, was from BD Biosciences (San Jose, CA, United States). Guinea pig anti-vesicular glutamate transporters type 1 antibody was from Millipore (Temecula, CA, United States). Guinea pig anti-VGAT was from AlomoneLabs (Jerusalem, Israel). Bradford assay was from Bio-Rad (Segrate, Milan, Italy). KAPA Mouse Genotyping Kits were from Kapa Biosystems (Woburn, MA, United States). ECL Advance^TM^ was from Amersham Biosciences (Piscataway, NJ, United States).

## Results

### Mouse Cortical GABAergic Nerve Endings Possess mGlu1, GABA_B1_, and GABA_B2_ Receptor Proteins

**FIGURE 1:**
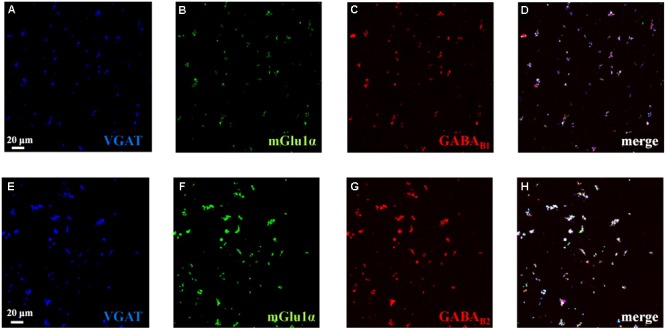
Identification and colocalization (white, **D** and **H**, merge) of GABA_B1_ and of GABA_B2_ and of mGlu1 receptor proteins and vesicular GABA transporter (VGAT) in mouse cortical synaptosomal particles. GABAergic synaptosomes were identified as VGAT-immunopositive particles (blue, panels **A** and **E**) and they were analyzed for the GABA_B1_ receptor protein content (red, panel **C**), for the GABA_B2_ receptor protein content (red, panel **G**) and for the mGlu1α receptor protein content (green, panels **B** and **F**). The figure shows representative images of five independent experiments carried out in different days.

We performed confocal analysis to detect the presence of mGlu1α, GABA_B1_, and GABA_B2_ immunopositivity in purified cortical synaptosomes that express the VGAT protein, here used as a selective marker of GABAergic particles. We identified a large colocalization (93 ± 3%) of GABA_B1_ (red, **Figure 1C**) or of GABA_B2_ (89 ± 2%) immunostaining (**Figure 1G**, red) with VGAT in hippocampal particles (blue, **Figures 1A,E**, respectively). Furthermore, VGAT-positive cortical synaptosomes (blue, **Figures 1A,E**, respectively) efficiently stained for mGlu1α receptor protein (green, **Figures 1B,F**, 66 ± 3%). Finally, we analyzed the colocalization of mGlu1α receptor protein (green, **Figures 1B,F**) with GABA_B1_ and GABA_B2_ subunits (red, **Figures 1C,G**, respectively). A diffuse colocalization of mGlu1α immune-positivity with GABA_B1_ and with GABA_B2_ subunit proteins was observed. The impossibility to perform a triple-labeling quantification, however, does not allow speculating the percentage of colocalization of the mGlu1 and GABA_B_ receptor proteins in the GABAergic synaptosomal particles, although there is a high overlapping of the mGlu1α with either the GABA_B1_ and GABA_B2_ stainings in the VGAT-positive particles (**Figures 1D,H**, merge, white).

### Presynaptic Release-Regulating GABA_B_ Autoreceptors and mGlu1 Heteroreceptors Functionally Interact in Mouse Cortical GABAergic Nerve Endings

Presynaptic release-regulating GABA_B_ autoreceptors exist in both rat (Pittaluga et al., 1987) and mouse (Lin et al., 1995) cortical synaptosomes. The activation of these receptors hampers the [^3^H]GABA exocytosis elicited by 12 mM KCl. Accordingly, 3 μM (±)baclofen significantly reduced the 12 mM KCl-evoked overflow of preloaded [^3^H]GABA from superfused mouse cortical synaptosomes (Bonanno et al., 1997). The (±)baclofen-induced effect was prevented by 0.1 μM of the GABA_B_ antagonist CGP52342 (12 mM KCl/3 μM (±)baclofen: 68.67 ± 3.47%; 12 mM KCl/3 μM (±)baclofen/0.1 μM CGP52342: 95.45 ± 4.56%, result expressed as percent of residual exocytosis, *n* = 5, *p* < 0.05, see also Raiteri, 2008). CGP52342 alone did not modify the KCl-evoked release of [^3^H]GABA from cortical synaptosomes (not shown).

**FIGURE 2:**
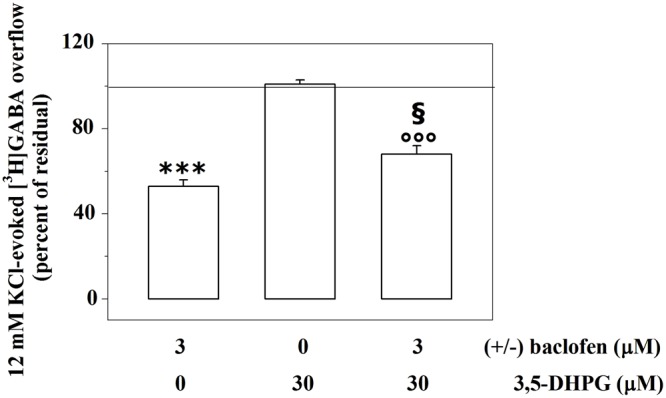
Presynaptic release-regulating GABA_B_ autoreceptors and mGlu1 heteroreceptors functionally cooperate to control GABA exocytosis from mouse cortical GABAergic nerve endings. Effects of 3 μM (±)baclofen and 30 μM 3,5-DHPG alone or concomitantly added on the 12 mM KCl-induced [^3^H]GABA overflow from mouse cortical nerve terminals. Results are expressed as percentage of the 12 mM KCl-induced [^3^H]GABA overflow (percent of residual). Data are the means ± SEM of five experiments run in triplicate. ^∗∗∗^*p* < 0.001 versus the 12 mM KCl-induced tritium overflow; ^°°°^*p* < 0.001 versus the 12 mM KCl/30 μM 3,5-DHPG-induced tritium overflow; ^§^ *p* < 0.05 versus the 12 mM KCl/3 μM (±)baclofen-induced tritium overflow.

**Figure 2** shows that the broad spectrum group I agonist 3,5-DHPG (30 μM, i.e., a drug concentration able to fully activate mGlu1 receptor subtypes, Musante et al., 2008) does not affect the 12 mM KCl-evoked release of the radiolabelled transmitter from cortical synaptosomes.

**FIGURE 3:**
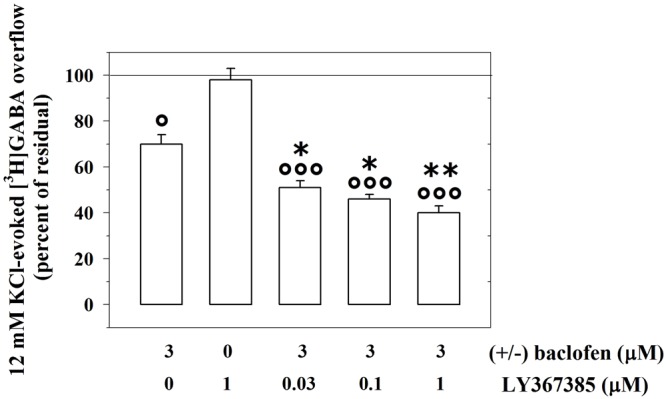
The mGlu1 receptor antagonist LY367385 favors the GABA_B_ autoreceptor-mediated control of preloaded [^3^H]GABA from mouse cortical GABAergic nerve endings. Effect of 3 μM (±)baclofen in the absence or in the presence of LY367385 (0.03–1 μM) on the release of preloaded [^3^H]GABA elicited by 12 mM KCl. Results are expressed as percentage of the 12 mM KCl-induced [^3^H]GABA overflow (percent of residual). Data are the means ± SEM of seven experiments run in triplicate. °*p* < 0.05 versus the 12 mM KCl-induced tritium overflow; ^∘∘∘^*p* < 0.001 versus the 12 mM KCl-induced tritium overflow; ^∗^*p* < 0.05 versus the 12 mM KCl/3 μM (±)baclofen-induced tritium overflow; ^∗∗^*p* < 0.01 versus the 12 mM KCl/3 μM (±)baclofen-induced tritium overflow.

We asked whether activating or inactivating mGlu1 ligands could modulate the release-regulating activity of presynaptic GABA_B_ autoreceptors. To this aim, experiments were carried out to quantify the impact of the mGlu1 receptor agonist 3,5-DHPG on the (±)baclofen-induced inhibition of [^3^H]GABA exocytosis. The mGlu1 agonist slightly, although significantly, reduced the 3 μM (±)baclofen-induced inhibition of the 12 mM KCl-evoked [^3^H]GABA overflow from superfused mouse cortical synaptosomes (**Figure 2**). Conversely, the mGlu1 antagonist LY367385 (0.03–1 μM) significantly amplified the inhibitory effect exerted by 3 μM (±)baclofen on the 12 mM KCl-evoked exocytosis of [^3^H]GABA. At the maximal concentration applied, the mGlu1 antagonist failed to affect on its own the release of tritium evoked by high KCl (**Figure 3**).

### Impact of *Grm1* Mutation on the GABA_B_ Autoreceptors Controlling GABA Release in Mouse Cortical GABAergic Nerve Endings

We then examined the 12 mM KCl-evoked overflow of preloaded [^3^H]GABA and its modulation by presynaptic release-regulating GABA_B_ autoreceptors in cortical synaptosomes from *Grm1^crv4/crv4^* mice, the mouse mutants bearing a genetic mutation that inactivate the mGlu1 receptor coding gene (*Gmr1*, Conti et al., 2006; Bossi et al., 2018). The release of [^3^H]GABA elicited by the mild depolarizing stimulus was not affected by the genetic mutation (WT mice, 12 mM KCl-evoked [^3^H]GABA overflow: 5.19 ± 0.53, *n* = 6; *Grm1^crv4/crv4^* mice, 12 mM KCl-evoked [^3^H]GABA outflow: 5.13 ± 0.48, *n* = 6; n.s.; data expressed as KCl-evoked tritium overflow).

**FIGURE 4:**
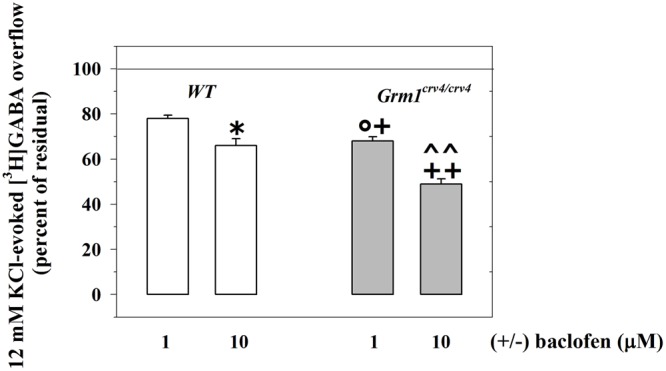
The mGlu1 genetic mutation affect the release-regulating activity of presynaptic GABA_B_ autoreceptors controlling [^3^H]GABA exocytosis from cortical GABAergic nerve endings. Synaptosomes from the cortices of control (*WT*, white bar) animals and of *Grm1^crv4/crv4^* mice (gray bar) were exposed to 12 mM KCl in the absence or in the presence of (±)baclofen (1–10 μM). Results are expressed as percentage of the 12 mM KCl-induced [^3^H]GABA overflow (percent of residual). Data are the media ± SEM of five experiments run in triplicate (three superfusion chambers for each mouse strain). ^∗^*p* < 0.05 versus the 12 mM KCl-evoked tritium overflow from *WT* cortical synaptosomes; ^+^*p* < 0.05 versus the 12 mM KCl-evoked tritium overflow from *Grm1^crv4/crv4^* cortical synaptosomes; ^++^*p* < 0.01 versus the 12 mM KCl-evoked tritium overflow from *Grm1^crv4/crv4^* cortical synaptosomes; °*p* < 0.05 versus the 12 mM KCl/1 μM (±)baclofen-evoked tritium overflow from *w.t.* cortical synaptosomes; ˆ ˆ *p* < 0.01 versus the 12 mM KCl/10 μM (±)baclofen-evoked tritium overflow from *w.t.* cortical synaptosomes.

Differently the inhibition of the [^3^H]GABA exocytosis elicited by (±)baclofen (1–10 μM) was significantly reinforced in *Grm1^crv4/crv4^* mouse cortical synaptosomes when compared to *w.t*. mice (**Figure 4**).

### Impact of the *Grm1* Mutation *crv4* on the GABA_B1_ and GABA_B2_ Receptor Proteins Expression in Mouse Cortical Nerve Endings

**FIGURE 5:**
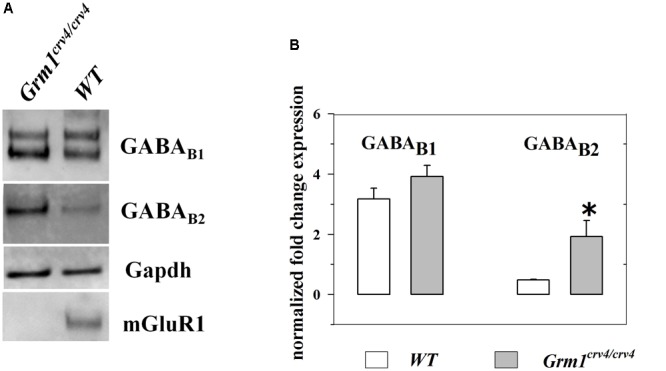
Expression of GABA_B2_ receptor proteins is significantly increased in cortical synaptosomes of *Grm1^crv4/crv4^* mice. Western blotting analyses were performed to determine the levels of GABA_B1_ and GABA_B2_ receptor expression in cortical synaptosomes (*n* = 4) of *Grm1^crv4/crv4^*, and age-matched *WT* mice. **(A)** Examples of immunoreactive bands obtained from cortical synaptosomes (20 μg proteins/lane) from *WT* and mutated mice. **(B)** Quantification of GABA_B1_ and GABA_B2_ receptor expression in cortical synaptosomes. The relative expression level of GABA_B1_ and GABA_B2_ receptor is expressed as the ratio of GABA_B1_ and GABA_B2_ receptor to the glyceraldeide-3-phosphate dehydrogenase (*Gapdh*) protein. Data represent the mean ± SEM (percentage *versus WT* mice). ^∗^*p* < 0.05 versus *WT* cortical synaptosomes.

According to results obtained from release experiments, an altered expression of GABA_B1_ receptor, GABA_B2_ receptor or both could account for functional changes in GABA_B_-mediated control of GABA exocytosis. Thus, we quantified the amount of GABA_B1_ and GABA_B2_ subunit proteins in cortical synaptosomes isolated from *Grm1^crv4/crv4^* and WT mice. **Figure 5** shows that the *Grm1* inactivating mutation did not cause a significant change in the GABA_B1_ subunit content when compared to WT mice. Differently, a significant enhancement of the amount of GABA_B2_ subunit was observed in *Grm1^crv4/crv4^* mice lacking mGlu1 receptors when compared to WT mice.

### PKC-Dependent Intraterminal Pathway Links mGlu1 and GABA_B_ Receptors in Mouse Cortical GABAergic Nerve Endings

**FIGURE 6:**
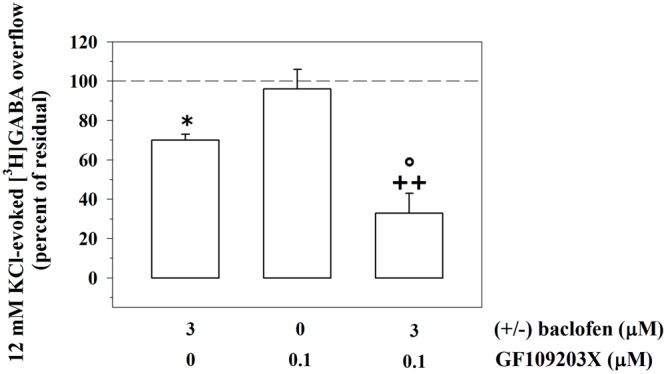
The release-regulating activity of presynaptic GABA_B_ autoreceptors in cortical GABAergic nerve endings depends on PKC-mediated intraterminal processes. Effects of the PKC inhibitor GF109203X (0.1 μM) on the 12 mM KCl in the absence or in the presence of 3 μM (±)baclofen. Results are expressed as percentage of the 12 mM KCl-induced [^3^H]GABA overflow (percent of residual). Data are the means ± SEM of four experiments run in triplicate. ^∗^*p* < 0.05 versus the 12 mM KCl-induced tritium overflow; ^++^*p* < 0.01 versus the 12 mM KCl/0.1 μM GF109203X- induced tritium overflow; ^°^*p* < 0.05 versus the 12 mM KCl/3 μM (±)baclofen-induced tritium overflow.

GABA_B_ receptors desensitize and desensitization often occurs because of an enhanced phosphorylation of the GABA_B_ proteins themselves. In particular, PKC activity attenuates the release-regulating activity of GABA_B_ receptors by promoting receptor desensitization, through the phosphorylation of the GABA_B1_ subunits and the dissociation from the *N*-ethylmaleimide-sensitive fusion (NSF) protein (Terunuma et al., 2010 and references therein). Activation of mGlu1 receptors triggers PKC-dependent phosphorylative pathways. We asked whether the mGlu1-mediated modulation of the GABA_B_-induced inhibition of GABA release relies on PKC-mediated events. To this aim, synaptosomes were exposed to GF109203X (0.1 μM), a PKC selective blocker, and the impact of 3 μM (±)baclofen on the 12 mM KCl-evoked [^3^H]GABA overflow was analyzed. **Figure 6** shows that the concomitant presence of the PKC inhibitor caused a huge significant reinforcement of the 3 μM (±)baclofen-induced inhibition of tritium overflow when compared to control condition. The PKC blocker failed to affect on its own the 12 mM KCl-evoked [^3^H]GABA exocytosis.

### Presynaptic Release-Regulating GABA_B_ Heteroreceptors and mGlu1 Autoreceptors Functionally Interact in Mouse Cortical Glutamatergic Nerve Endings

**FIGURE 7:**
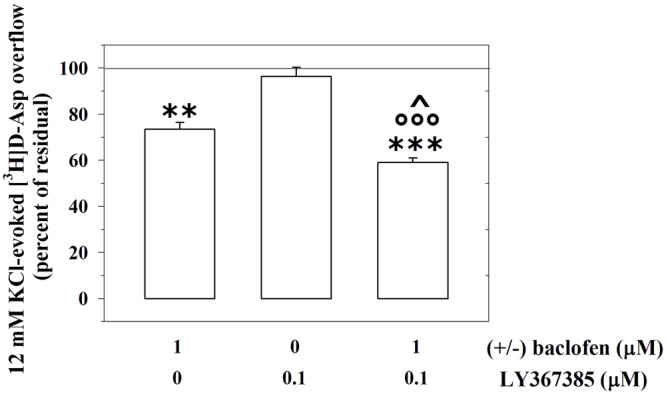
The mGlu1 receptor antagonist LY367385 amplifies the GABA_B_ heteroreceptor-mediated control of [^3^H]D-aspartate release from glutamatergic mouse cortical nerve endings. Effect of 1 μM (±)baclofen in the absence or in the presence of LY367385 (0.1 μM) on the release of preloaded [^3^H]D-aspartate ([^3^H]D-Asp) elicited by 12 mM KCl. Results are expressed as percentage of the 12 mM KCl-induced [^3^H]D-Asp overflow (percent of residual). Data are the means ± SEM of five experiments run in triplicate. ^∗∗^*p* < 0.01 versus the 12 mM KCl-induced tritium overflow; ^∗∗∗^*p* < 0.001 versus the 12 mM KCl-induced tritium overflow; ^∘∘∘^*p* < 0.001 versus the 12 mM KCl/0.1 μM LY367385-induced tritium overflow; ˆ*p* < 0.05 versus the 12 mM KCl/1 μM (±)baclofen-induced tritium overflow.

We asked whether the mGlu1-GABA_B_ receptor–receptor interaction is restricted to the GABAergic nerve endings or, alternatively, if it also occurs in other subpopulations of nerve endings. Presynaptic release-regulating GABA_B_ heteroreceptors exist in cortical glutamatergic terminals. By acting at these receptors, (±)baclofen, inhibits significantly the 12 mM KCl-induced [^3^H]D-Aspartate ([^3^H]D-Asp) exocytosis (Raiteri, 2008). Accordingly, 1 μM (±)baclofen significantly reduced the 12 mM KCl-evoked overflow of preloaded [^3^H]D-Asp (**Figure 7**). Furthermore, these terminals also possess mGlu1 autoreceptors, whose activation potentiates the 12 mM KCl-evoked release of [^3^H]D-Asp (Musante et al., 2008). **Figure 7** shows that, when concomitantly added to 1 μM (±)baclofen, LY367385 (0.1 μM) significantly reinforced the inhibitory effect exerted by the GABA_B_ agonist on the 12 mM KCl-evoked overflow of preloaded [^3^H]D-Asp. At the concentration applied LY367385 did not modify the release of tritium evoked by high KCl (see also Musante et al., 2008).

### mGlu1, GABA_B1_, and GABA_B2_ Receptor Proteins Colocalize in Mouse Cortical Glutamatergic Nerve Endings

**FIGURE 8:**
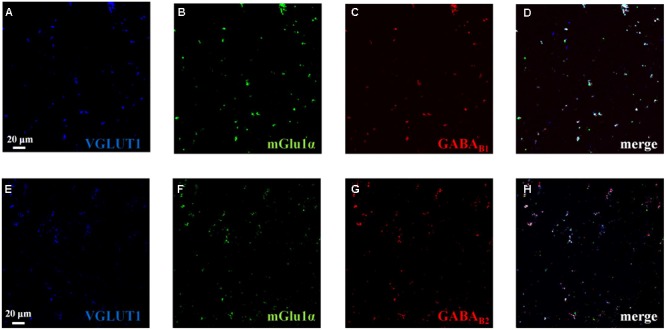
Identification and colocalization (white, **D** and **H**, merge) of GABA_B1,_ of GABA_B2_ and of mGlu1 receptor proteins and of VGLUT1 in mouse cortical synaptosomal particles. Glutamatergic synaptosomes were identified as VGLUT1-immunopositive particles (blue, panels **A** and **E**) and they were analyzed for the GABA_B1_ receptor protein content (red, panel **C**), for the GABA_B2_ receptor protein content (red, panel **G**) and for the mGlu1 receptor protein content (green, panels **B** and **F**). The figure shows representative images of four independent experiments carried out in different days.

Confocal microscopy was also performed by labeling cortical synaptosomes with VGLUT1 antibody (blue, **Figures 8A,E**), to highlight glutamatergic nerve endings, and with anti-mGlu1α antibody (green, **Figures 8B,F**) and with antibodies recognizing the GABA_B1_ (red, **Figure 8C**) and the GABA_B2_ (green, **Figure 8G**) receptor proteins. Synaptosomal preparations efficiently stained for all the antibodies tested and the colocalizations of VGLUT1 and mGlu1α receptor proteins, of VGLUT1 and GABA_B1_ receptor proteins and of VGLUT1 and GABA_B2_ receptor protein were analyzed. Merging of the appropriate image pairs revealed that a large percentage of VGLUT1-positive particles expressed mGlu1 receptor proteins (46 ± 7%), as well as GABA_B1_ (52 ± 7%) and GABA_B2_ (45 ± 6%) receptor subunits. Confocal analysis also unveiled a colocalization of mGlu1α staining with GABA_B1_ and GABA_B2_ immuno-positivities. Again, the triple-labeling quantification of the percentage of colocalization of the mGlu1 and GABA_B_ receptor proteins in the glutamatergic synaptosomes cannot be proposed. However, the merged images (**Figures 8D,H**, merge, white) indicates a high overlapping of the mGlu1α with either the GABA_B1_ and GABA_B2_ stainings in the VGLUT1-positive particles.

## Discussion

The existence and the role of mGlu1 receptors controlling presynaptically the release of GABA has been argument of discussion for several years. GABAergic interneurons were proposed to possess mGlu1 receptors the activation of which alters inhibitory transmission, also at GABAergic autapses. This conclusion was also supported by the observations that mGlu1 antagonists are neuroprotectant and that neuroprotection is abolished by increasing the [GABA]_out_ by means of GABA uptake inhibitors (Baude et al., 1993; Gereau and Conn, 1995; Morishita et al., 1998; Battaglia et al., 2001). Most of these studies, however, failed to prove the existence of presynaptic release-regulating mGlu1 receptors in GABAergic nerve endings. Rather, some of them proposed the existence of postsynaptic mGlu1 receptors promoting the endogenous production of cannabinoids that retrogradally modulate GABA release (Alger, 2002; Diana et al., 2002; Gerdeman et al., 2002; Pellegrini-Giampietro, 2003; Ferraguti et al., 2008). The complexity of the scenario is now further implemented by our results which suggest the existence of presynaptic mGlu1 receptors coupled in an “antagonist-like manner” to presynaptic GABA_B_ receptors.

Neurotransmitters are usually analyzed individually, for their releasing activity, unmindful that they also trigger receptor-mediated events that control the function(s) of other, by-standing, receptors. The complexity that originates from these converging actions is referred to as “metamodulation” and have a huge impact on synaptic transmission in CNS (Marchi et al., 2015). A useful approach to study “metamodulation” is the technique of the “*up-down superfusion of a thin layer of synaptosomes*” (Raiteri et al., 1974; Raiteri and Raiteri, 2000; Pittaluga, 2016). By assuring the rapid removal of any substances endogenously released, this technique prevents the presence of the biophase, then impeding the onset of indirect events due to endogenous compounds acting at presynaptic receptors (including those produced postsynaptically, i.e., the endocannabinoids). This approach represents therefore a method of choice to highlight the functional cross-talk linking presynaptic receptors (Pittaluga et al., 2000, 2005; Musante et al., 2011; Summa et al., 2011; Grilli et al., 2012; Di Prisco et al., 2016). By a functional point of view, the receptor–receptor interaction can be evidentiated in release studies as change(s) in transmitter release efficiency observed when exposing concomitantly synaptosomes to exogenous ligands acting at the colocalized receptors (Longordo et al., 2006; Luccini et al., 2007; Olivero et al., 2018). In general, it is proposed that two receptors coexist and functional couple when the releasing activity due to their concomitant activations differs quantitatively from the sum of the releasing effects elicited by each receptor (Pittaluga and Raiteri, 1992).

The fact that mGlu1 ligands cannot modify GABA exocytosis (Pittaluga and Raiteri, 1992; Musante et al., 2010; Zucchini et al., 2013) hugely simplifies the system and led us to propose the cortical GABAergic terminals as an appropriate model to investigate the consequences of the mGlu1/GABA_B_ receptor–receptor interaction. In release studies, we confirmed that neither 3,5-DHPG nor LY367385 caused changes to GABA exocytosis. The two ligands, however, significantly influenced the control of GABA exocytosis elicited by presynaptic GABA_B_ autoreceptors. In particular, the mGlu1 agonist significantly reduced the (±)baclofen-mediated inhibition of GABA exocytosis, while the orthosteric mGlu1 selective antagonist reinforced it. On the basis of the above considerations, these observations were predictive of the existence of presynaptic mGlu1 heteroreceptors on GABAergic nerve endings and of their functional cross-talk with GABA_B_ autoreceptors.

The efficacy of the orthosteric antagonist in controlling GABA_B_-mediated signaling deserves some comments. The lack of biophase makes unlike the possibility that the mGlu1 antagonist can compete with the endogenous glutamate for the binding at the presynaptic mGlu1 heteroreceptors on GABAergic terminals. The possibility, however, exists that, in cortical synaptosomes, mGlu1 heteroreceptors could have adopted a constitutive active conformation (De Blasi et al., 2001). This conformation would assure a productive coupling of the receptor to the associate G protein and the propagation of the mGlu1-mediated signaling, despite the absence of the agonist in the biophase (Musante et al., 2008; Rossi et al., 2013). If this is the case, the binding of the orthosteric antagonist would force a conformational change of the receptor protein, interrupting the coupling of mGlu1 receptors with the G proteins and the associated intraterminal cascade of events that, we speculate, could reverberate on the co-localized GABA_B_ receptor protein (see below).

Interestingly, the genetic deletion of mGlu1 receptor proteins affects the (±)baclofen-mediated inhibition of GABA exocytosis in a way that is reminiscent of the impact of mGlu1 antagonist on the GABA_B_ receptor. In particular, the GABA_B_-mediated inhibition of GABA release in cortical nerve endings from *Grm1^crv4/crv4^* mice is more efficient when compared to *WT* mice, but it is largely comparable to that observed in the presence of the mGlu1 orthosteric antagonist. To note, in cortical synaptosomes from mutant mice, the expression of the GABA_B2_ receptor subunit, i.e., the subunit that dictates the affinity of (±)baclofen at GABA receptor (Møller et al., 2017 and references therein), is largely increased when compared to *WT* mice. This observation could give the rationale for the changes in the agonist efficacy observed in mutant mice; further studies are required to correctly address this point.

To summarize, the observations depicted so far seem best interpreted by assuming that: (i) mGlu1 heteroreceptors exist in GABAergic terminals; (ii) they colocalize with GABA_B_ autoreceptors; (iii) the activation of mGlu1 receptors influences the GABA_B_-mediated control of GABA exocytosis; (iv) the genetic deletion of mGlu1 receptors affect the expression and the presynaptic release-regulating activity of GABA receptors.

The second result of our study is that the “mGlu1 to GABA_B_” receptor-receptor cross–talk is not restricted to GABAergic nerve endings, but rather represents a wide-spread event that occurs also in other subfamilies of cortical nerve endings, i.e., the glutamatergic ones. Actually, also in cortical glutamatergic synaptosomes, the blockade of the presynaptic mGlu1 autoreceptors reinforced the inhibitory tune exerted by (±)baclofen at inhibitory presynaptic GABA_B_ heteroreceptors.

In both synaptosomal populations, the “enabling” modulatory effect exerted by mGlu1 antagonists on GABA_B_ receptors occurs because of the co-existence of the two receptors on the same nerve endings, as confirmed by confocal microscopy showing the overlapping of mGlu1α, GABA_B1_, and GABA_B2_ immunostainings in either the VGAT-containing or the VGLUT1-positive synaptosomal particles. For the sake of clarity, the mGlu1/GABA_B_ receptor–receptor interaction was already reported in the literature. In particular, in Purkinje cells, a GABA_B_-mediated control of mGlu1-induced signaling was described, which relied on the physical association of mGlu1 receptor protein with the GABA_B_ receptor complex. The assembly of the receptor complex was independent on G protein-mediated mechanisms, but dependent on external calcium ions. The final outcome was an increased sensitivity of mGlu1 receptors to glutamate (Dittman and Regehr, 1996, 1997; Vigot and Batini, 1997; Tabata et al., 2004). Unfortunately, in the present case, the role of external calcium in dictating the mGlu1-GABA_B_ receptor–receptor interaction cannot be investigated, since the removal of the cation from the superfusion medium abrogates *per se* the transmitter exocytosis.

On the basis on the main features of the receptor(s) involved in the receptor-receptor cross-talk, some speculations on the molecular event(s) underlying the receptor-receptor cross-talk can be proposed. It is known that activation of mGlu1 receptors preferentially leads to the translocation of phospholipase C, hydrolysis of membrane phosphoinositide and accumulation of diacylglycerol and calcium ions in the cytosol, which in turn activate PKC-mediated processes, including GABA_B_ receptor desensitization. Actually, the phosphorylation of the carboxy terminus of the GABA_B1_ subunit assure its dissociation from the NSF protein and the desensitization of the receptor (Terunuma et al., 2010). Blockade of the PKC-dependent phosphorylative processes should therefore be expected to slow GABA_B_ receptor desensitization, reinforcing its inhibitory control on transmitter exocytosis, as indeed observed with the selective PKC blocker GF109209X. On the basis of these considerations, we propose that the mGlu1-GABA_B_ receptor-receptor cross-talk involves a PKC-dependent intraterminal phosphorylative pathway which modulate GABA_B_ receptor desensitization.

## Conclusion

The results from our study highlight a functional cross-talk linking excitatory glutamatergic receptors (the mGlu1 receptor subtype) and inhibitory GABAergic receptor (the GABA_B_complex). We provide evidence that the two receptors colocalize in both glutamatergic and GABAergic terminals and that the mGlu1 receptors tune in an antagonist-like manner the efficiency of the presynaptic release-regulating GABA_B_ receptors.

Synaptic efficiency depends on the equilibrium between the excitatory and the inhibitory inputs on neurons. mGlu1 antagonist or negative mGlu1 allosteric modulator (NAM) would reinforce the paracrine effect of GABA at GABA_B_ heteroreceptors located on glutamatergic nerve endings, positively tuning the excessive glutamate release that often characterize central neurological diseases. Concomitantly, mGlu1 antagonist and mGlu1 NAM would reduce GABA exocytosis from GABAergic terminals, because of the reinforcement of the inhibitory tone of GABA_B_ autoreceptors at this level. The “enhancement” of the paracrine GABAergic control of glutamate release from nerve endings would compensate for the diminished spillover of GABA at GABA_B_ heteroreceptors Although further studies are required to define the impact of “*in vivo*” administration of mGlu1 ligands on the presynaptic GABA_B_-mediated control of both transmitters, our findings improve the knowledge of the complex homeostatic mechanisms of control of the excitatory/inhibitory balance in CNS.

## Author Contributions

APi designed the experiments, supervised the execution of the research activity and the statistical analysis, and wrote the manuscript. GO, MV, and FC performed release experiments. CU performed confocal microscopy. APu and SB made available to the study the *Grm1^crv4/crv4^* mice, and performed genotyping of these animals and Western blot analysis in both *Grm1^crv4/crv4^* and *WT* mice. GO, MV, FC, SB, APu, and CU approved the final version of the manuscript and agreed to be accountable for all the aspects of the work.

## Conflict of Interest Statement

The authors declare that the research was conducted in the absence of any commercial or financial relationships that could be construed as a potential conflict of interest.
